# Reading between the lines: exploring the discriminative ability of the Short-Story Task in identifying autistic individuals within autism outpatient services

**DOI:** 10.3389/fpsyt.2025.1500396

**Published:** 2025-02-04

**Authors:** Irina Jarvers, Monika Sommer, Manuela Ullmann, Verena Simmel, Lore Blaas, Stefanie Gorski, Saskia Krüger-Lassen, Matthias Vogel, Berthold Langguth

**Affiliations:** ^1^ Department of Child and Adolescent Psychiatry and Psychotherapy, University of Regensburg, Regensburg, Germany; ^2^ Department of Psychiatry and Psychotherapy, University of Regensburg at the Bezirksklinikum Regensburg, Regensburg, Germany; ^3^ Department of Psychology, Ludwig-Maximilians-University of Munich, Munich, Germany

**Keywords:** Short-Story Task, autism outpatient services, fiction-based mentalizing, diagnostics, autism spectrum

## Abstract

**Introduction:**

The correct diagnosis of autistic individuals is an everyday challenge within autism outpatient services. While the short-story task (SST), a task measuring fiction-based mentalizing, has demonstrated promise in differentiating between autistic and non-autistic adults, its discriminative ability has not been investigated in a sample of individuals seeking autism diagnostics at outpatient services.

**Methods:**

This study aimed to evaluate the utility of the SST in individuals seeking autism diagnostics between 2016 and 2022 at the Clinic and Polyclinic for Psychiatry & Psychotherapy of the University of Regensburg at medbo District Hospital Regensburg. The sample consisted of 211 individuals. In 100 of them an autism spectrum disorder has been diagnosed and 111 individuals were diagnosed with other conditions or none at all.

**Results:**

Performance on the SST did not significantly differ between the two groups, and receiver operator curve analysis did not support the SST as a reliable discriminator. However, linear regression analyses revealed that autism diagnosis was the sole significant predictor of SST mentalizing performance. Additionally, specific items of the SST showed significant differences between autistic and non-autistic individuals and constituted a significant predictor of autism diagnosis.

**Discussion:**

While the SST may not be robust enough to accurately identify autistic individuals on its own, it does offer clinicians valuable insights into how individuals interpret others’ actions and whether they grasp the broader context of a story versus focusing solely on details.

## 1 Introduction

Autism Spectrum Disorder (ASD) is a lifelong neurodevelopmental condition characterized by a range of differences in social communication, behavior, and sensory processing (1). An accurate diagnosis of autism is paramount for several reasons. Individuals who are formally diagnosed with autism can receive specific support that significantly improves their quality of life (2). For instance, once diagnosed, individuals can secure tailored educational plans that cater to their learning styles and needs, and obtain workplace accommodations that enhance their job performance and job satisfaction (3). This support directly addresses the unique challenges faced by autistic individuals, promoting better educational and occupational outcomes. Furthermore, a diagnosis can foster a sense of community and belonging. Studies have shown that autistic individuals who connect with others in the autistic community experience lower levels of loneliness and increased emotional support (3, 4).

Beyond post diagnostic support and establishing a community, an accurate diagnosis can validate the struggles and experiences that individuals have faced, sometimes for many years (3). This validation contributes to enhanced self-acceptance and self-esteem, as individuals no longer feel as though they are struggling in isolation or grappling with unrecognized difficulties (5, 6). Research by Powell and Acker (7) indicates that a diagnosis can help individuals make sense of their experiences and behaviors, fostering a positive self-identity and reducing feelings of isolation or being misunderstood.

Overall, the above highlights the comprehensive benefits of receiving an autism diagnosis. However, the diagnostic process, especially in adulthood, is difficult. Autism’s heterogeneity means that autistic features can present very differently across individuals, making standardized diagnostic tools less effective (8). Furthermore, clinicians often struggle with limited resources and institutional pressures, which complicate the diagnostic process and can lead to missed or misdiagnoses (1). The complexity is compounded for adults who may have developed coping mechanisms, such as camouflaging their symptoms, making them appear non-autistic to others, even professionals (9).

A further significant challenge in diagnosing autism is the fact that other psychiatric disorders can mask or mimic autism symptoms. Many autistic individuals also experience additional psychiatric comorbidities, such as depression, anxiety, and attention deficit hyperactivity disorder (ADHD) (10–12). Jadav and Bal (13) found that psychiatric conditions are more frequently endorsed by adults diagnosed with autism in adulthood compared to those diagnosed in childhood. These comorbidities can complicate the diagnostic process, leading to misdiagnosis or delayed diagnosis (10).

Diagnosing autism in women presents another layer of difficulty. Research indicates that females are often diagnosed later than males and are more likely to be misdiagnosed initially (14). This delay and misdiagnosis can be attributed to a higher likelihood of women to camouflage their features, employing strategies to hide their autistic traits and better fit into social norms (9). This camouflaging behavior not only makes it harder for clinicians to identify autism but is also associated with worse mental health outcomes, as individuals exert significant effort to mask their true selves (15).

Additionally, alexithymia, a condition characterized by difficulty in identifying and describing emotions, is prevalent among autistic individuals and complicates the diagnostic process (16). Hobson et al. (17) found that alexithymia can affect the outcomes of autism diagnostic assessments, as socio-emotional abilities, which are critical for these assessments, are impaired. This can lead to an underestimation of autistic symptoms or even a failure to diagnose autism altogether.

Overall, while receiving an autism diagnosis is important and beneficial for autistic individuals, the diagnostic process is accompanied by many struggles, resulting in misdiagnoses that complicate prevalence data, affect research outcomes and may misdirect individuals from therapies that best suit their needs (18–20). To overcome these issues, it is essential to implement robust and comprehensive diagnostic practices that ensure individuals receive the appropriate support and intervention. One reason for these difficulties is the lack of well-validated measures that accurately capture the real-life social difficulties autistic individuals experience in daily interactions with non-autistic individuals. This highlights the critical need to safeguard research integrity and optimize resource allocation by ensuring diagnostic tools are reliable, valid, and reflective of the complexities of autism. Addressing this diagnostic gap requires the development and use of more nuanced tools that can improve diagnostic accuracy and reduce the risk of misdiagnosis.

One promising tool that has emerged recently is the Short Story Task (SST) (21). In the SST, participants read ‘The End of Something’ by Ernest Hemingway and answer a series of comprehension, and mentalizing questions. This story-like design closely mirrors real-life experiences and assesses the difficulties autistic individuals face in interpreting the actions of non-autistic individuals. The SST has demonstrated discriminatory power when differentiating between autistic and non-autistic individuals (22), as well as distinguishing autistic individuals from those with depression (23).

While these initial findings are promising, they primarily involve comparisons with healthy non-autistic individuals or those with isolated psychiatric conditions. They do not fully reflect the complexity of real-world clinical settings, where mentalization impairments are often observed across a range of psychiatric disorders (24). In outpatient services for autism diagnostics, patients are typically not compared to individuals without any mental health problems but rather to those experiencing various forms of psychological distress and social difficulties (25). These individuals seek help for challenges they face in daily life, often suspecting autism as a possible underlying cause. This creates a more nuanced and challenging diagnostic landscape, where tools like the SST must demonstrate their reliability and specificity in distinguishing autism-related difficulties from those associated with other conditions.

To explore whether the SST can meet these challenges, we conducted a retrospective study involving 211 outpatients who presented at a specialized outpatient autism service at a tertiary referral center (Clinic and Polyclinic for Psychiatry and Psychotherapy at the University of Regensburg). Our objective was to evaluate the SST’s discriminatory power within this heterogeneous clinical population, focusing on its ability to differentiate between those ultimately diagnosed with autism and those who were not. We hypothesized that the SST would show sufficient discriminatory ability in this setting, providing a valuable tool for improving the accuracy and reliability of autism diagnostics in outpatient services.

## 2 Methods

### 2.1 Participants

Data of a total of 211 individuals was evaluated retrospectively (*M*_age_ = 31.08 years, *SD* = 11.18 years, range = 18–62). Among them, 100 were eventually diagnosed with autism spectrum disorder (ASD), and 111 were diagnosed with either a different clinical condition or no condition at all. All participants attended autism diagnostic sessions at the outpatient autism service of the Clinic and Polyclinic for Psychiatry & Psychotherapy at the University of Regensburg, located at medbo District Hospital Regensburg, between 2016 and 2022. Diagnostic sessions included standard measures for autism diagnostics in adulthood and the SST, which was subsequently evaluated to assess its specificity. The retrospective study was approved by the ethics committee of the University of Regensburg (Nr.: 22-3153-104) and is pre-registered in the German Clinical Trials Register (DRKS00033679).

### 2.2 Assessment

Demographic variables were coded via the patient information available in the digital patient record, including age, sex, education, relationship status and current employment status.

Participants’ IQ was assessed using the German version of the third edition of the Wechsler adult intelligence scale [WAIS-III (26); German translation: WIE (27)]. In the WAIS-III, IQ is assessed through Verbal IQ, Non-Verbal (Performance) IQ, and a Full-Scale IQ, which provides a comprehensive evaluation of an individual’s cognitive abilities. Verbal IQ is calculated using six subtests: Vocabulary, Similarities, Arithmetic, Information, Digit Span, and Comprehension. These subtests focus on language-based reasoning, general knowledge, memory, and understanding. Non-Verbal (Performance) IQ is determined by five subtests: Picture Completion, Digit Symbol-Coding, Block Design, Matrix Reasoning, and Picture Arrangement. These assess visuospatial abilities, pattern recognition, and problem-solving skills. The Full Scale IQ combines the scores from both Verbal and Non-Verbal IQ to offer an overall measure of intellectual functioning (28).

#### 2.2.1 The Short-Story Task

For the Short-Story Task (SST), we adhered to the protocol outlined by Dodell-Feder et al. (21) which was translated into German by Jarvers et al. (22). Participants were asked to read the German translation of “The End of Something,” rendered by E. Horschitz-Horst and A. Ceram, C. W., with a specific focus on the interactions between the two main characters. The story centers on a couple undergoing a breakup, triggered by the man’s waning interest in the relationship. The absence of explicit descriptions of the characters’ mental states makes the narrative particularly effective for eliciting mentalizing responses from participants.

After reading, participants were instructed to summarize the plot. If their summaries naturally included insights into the characters’ mental states, they were awarded one point; otherwise, they received zero points (spontaneous mentalizing). Following this, participants responded to four comprehension questions (e.g., “Nick and Marjorie have a pail of perch for what purpose?”), eight mentalizing questions (e.g., “What does Nick mean when he says, ‘It isn’t fun anymore’?”), and a final comprehension question (e.g., “The story is called ‘The End of Something.’ What is the title referring to?”). Each of these questions allowed participants to earn between 0 and 2 points, with a maximum of 10 points for comprehension and 16 points for mentalizing.

Given previous findings that the number of books read significantly influences SST performance (22), participants were asked to report the average number of fiction books they read per month. Responses were categorized as follows: zero books per month (0), less than one book per month (1), between one and two books per month (2), and more than two books per month (3).

#### 2.2.2 Additional diagnostic measures

In addition to the SST, autism diagnostics included the German versions of the Adult Asperger Assessment [AAA (29)] which includes the Autism Quotient [AQ (30)] and the Empathy Questionnaire [EQ (31)]. The AAA assesses various domains, including social and communication difficulties, repetitive behaviors, and narrow interests as a mixture of self-reported questionnaires and clinical interviews.

Additionally, alexithymia, the difficulty to identify and describe one’s own emotions, was assessed using the Toronto Alexithymia Questionnaire 26 [TAS-26 (32, 33)]. The TAS-26 is the only version of the TAS that is well-validated in German and has reliable cut-offs (< 51 for low alexithymia and >61 for high alexithymia). It includes three subscales: Difficulty Identifying Feelings (DIF), which measures how challenging it is for individuals to recognize their own emotions; Difficulty Describing Feelings (DDF), which assesses the difficulty in articulating feelings to others; and Externally-Oriented Thinking (EOT), which evaluates a focus on external events rather than internal emotional experiences (32).

Finally, the Sensory Inventory [“Sensorik Inventar” SI (34)] was administered, a German measure designed to assess sensory sensitivities. The SI is a specialized tool for evaluating sensory processing and integration, focusing on how individuals perceive and respond to various sensory stimuli. It includes subscales for Vision, Hearing, Smell/Taste, Touch, Pain/Temperature, and Multiple Stimuli, which can be combined to produce a total score. Additionally, the SI allows for the calculation of two separate scales: Sensory Seeking and Body Perception. For the total score, a difference score is computed based on age norms for men and women. This difference score is reported in the present study.

### 2.3 Procedure

After expressing interest in a diagnostic assessment for autism, patients completed and mailed several questionnaires, including the anamnesis form, EQ, AQ, TAS-26, and the sensory inventory. Ideally, they also provided supporting documents such as school records, the child health booklet, and prior medical reports. At the initial appointment at the autism outpatient clinic, both a physician and psychologist evaluated the patient. If autism spectrum disorder (ASD) is not ruled out during this appointment, additional psychological diagnostic sessions were scheduled (three sessions, each lasting 2 to 2.5 hours), and a phone call with a parent was arranged, if possible.

The psychological testing appointments were performed in the following sequence, with a brief 5–10-minute break after 60 minutes: WAIS-III (administered in two parts; the first part during the initial session), Short Story Task, WAIS-III (second part), and AAA-Interview. Following the testing, an external anamnesis is conducted via phone with a parent, without the patient being present. After all information has been gathered, the results were reviewed and discussed in a team meeting with the physician, leading to the preparation of the diagnostic report. The final step involved communicating the test results to the patient. Overall, patients attended five appointments at our facility. For this study, data from these assessment appointments, conducted between 2016 and 2022, were coded from both digital and analogue records and subsequently evaluated regarding the SST.

### 2.4 Statistical analysis

The data were analyzed using SPSS 29 (35). Statistical significance was set at α = 0.05 and multiple comparisons where controlled for using the false-discovery rate where appropriate (36).

First, Mann-Whitney U tests were performed to compare basic demographics and performance on autism measures, including the SST, between individuals with an autism diagnosis (ASC) and those with other or no diagnoses (no-ASC). Next, area under the curve (AUC) receiver operating characteristic (ROC) analyses were conducted to evaluate the SST’s discriminatory ability. AUC values were interpreted as follows: 0.50–0.70 indicated poor discrimination, 0.70–0.80 indicated acceptable discrimination, 0.80–0.90 indicated excellent discrimination, and values above 0.90 indicated superior discrimination (37).

Following the approach of Jarvers et al. (23), we conducted two regression analyses: (a) a multivariable regression to predict mentalizing performance using factors such as group membership (ASC vs. no-ASC), spontaneous mentalizing in the SST, SST comprehension, number of books read, verbal IQ, nonverbal IQ, and education; and (b) a binomial logistic regression to predict ASC group membership based on SST mentalizing and comprehension scores, number of books read, verbal IQ, nonverbal IQ, age, sex, and education.

Furthermore, we performed exploratory comparisons using χ² tests to identify significant group differences in responses to specific items on the SST. Finally, a second binomial logistic regression was conducted to predict group assignment, using the significant items identified in the group comparisons as predictors instead of the SST mentalizing score.

## 3 Results

**Figure 1:**
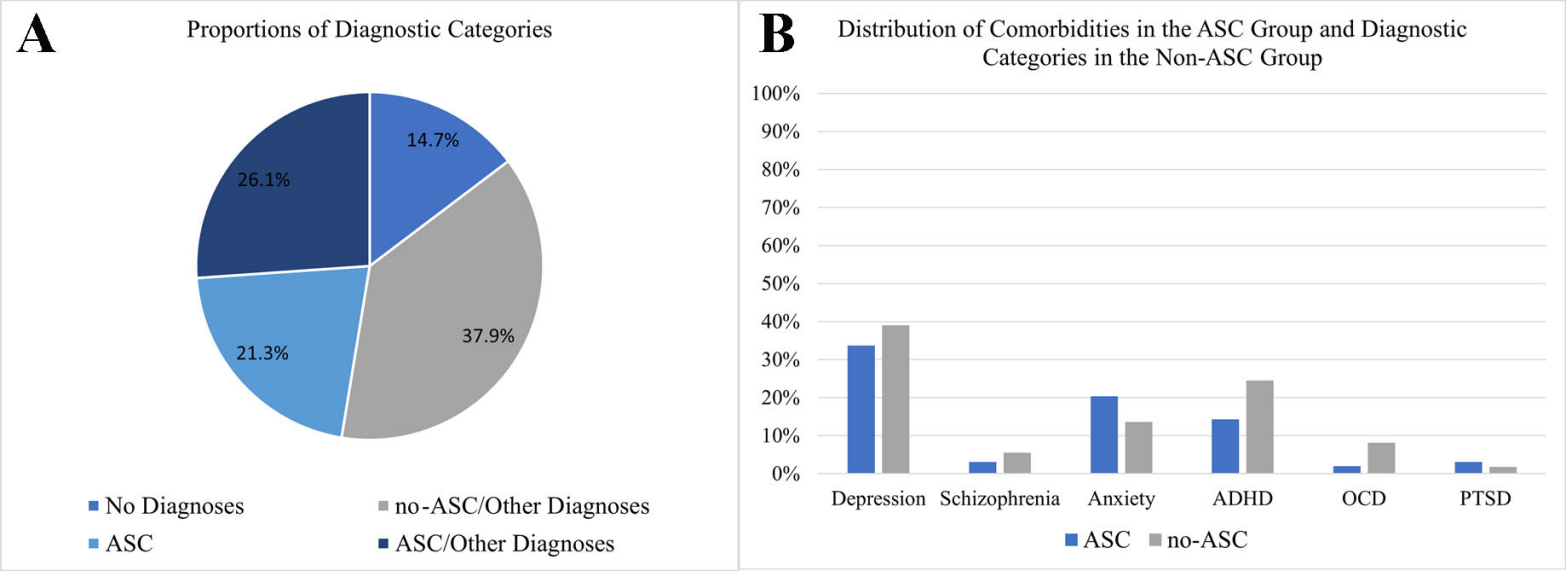
Overview of diagnoses and comorbidities in the sample. **(A)** A depiction of proportions of diagnostic categories, **(B)** an overview of the distribution of comorbidities in the ASC group and diagnostic categories in the no-ASC group. ASC, group of individuals diagnosed with autism; no-ASC, group of individuals not diagnosed with autism; ADHD, attention-deficit-hyperactivity disorder; OCD, obsessive-compulsive disorder; PTSD, post-traumatic stress disorder.

**Figure 2:**
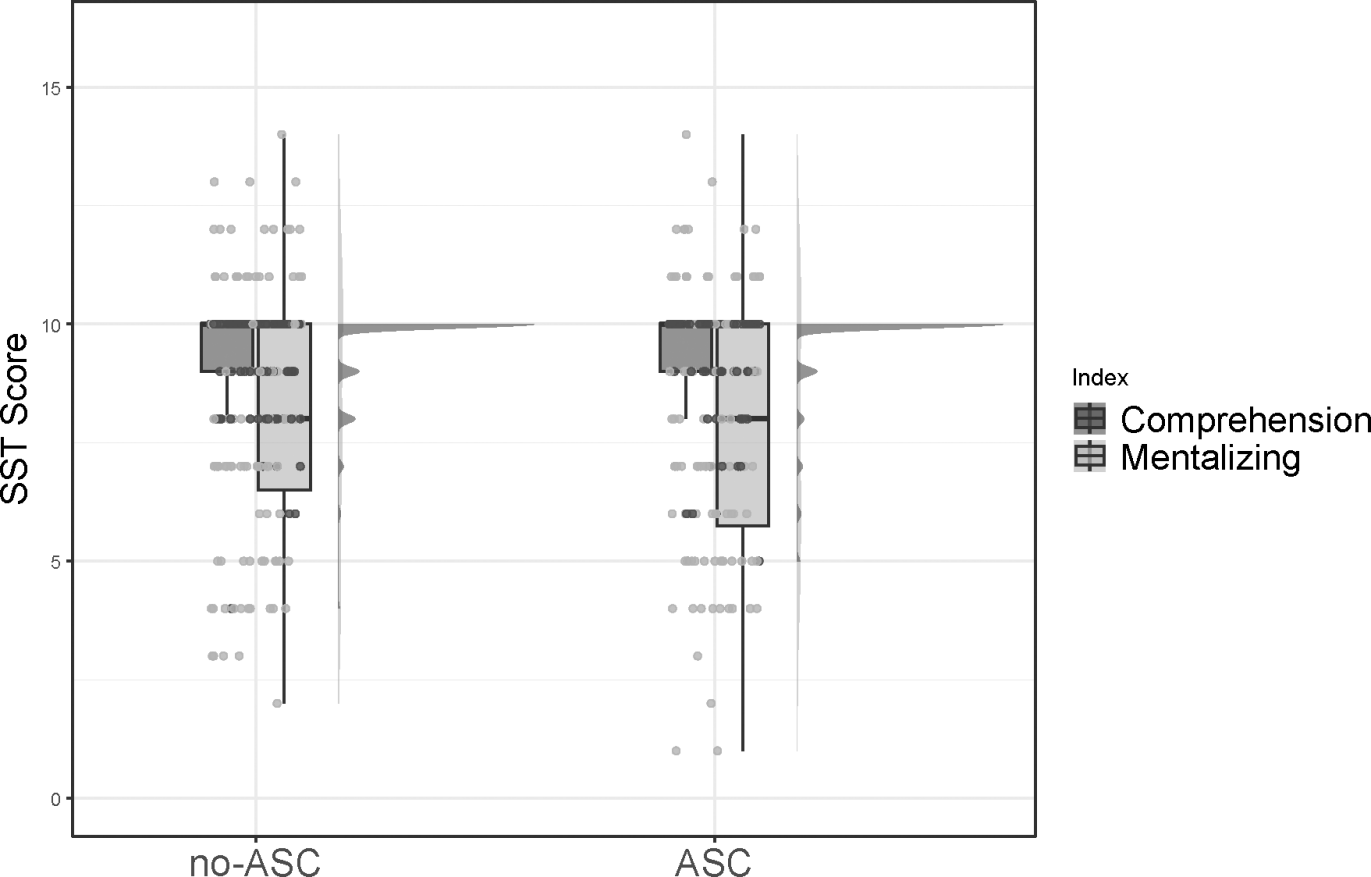
Raincloud plot depicting performance on the SST for the no-ASC group (left) and the ASC group (right). Performance is depicted separately for the comprehension scale and the mentalizing scale. ASC, group of individuals diagnosed with autism; no-ASC, group of individuals not diagnosed with autism.

Descriptive statistics for the total sample, as well as for the ASC and no-ASC groups, are presented in **Table 1**. There were no significant differences between the groups in sex, age at first contact, verbal IQ, non-verbal IQ, level of education, or total alexithymia score (see **Table 1**). However, the ASC group scored significantly higher on the EOT alexithymia subscale compared to the no-ASC group, indicating a higher level of externally oriented thinking. Furthermore, the groups did differ significantly in their scores on the AAA, AQ, EQ, and SI difference measure (see **Table 1**). Specifically, the ASC group exhibited higher scores on the AAA, AQ, and SI, but lower scores on the EQ compared to the non-ASC group. Regarding SST performance, no significant differences were found between the ASC and non-ASC groups in either comprehension or mentalizing (see **Table 1**). Finally, there was a significant difference in the number of books read, with the ASC group reporting fewer books read. The distribution of diagnostic categories and other psychiatric diagnoses across the two groups is depicted in **Figure 1**. See **Figure 2** for a graphical depiction of SST performance across groups.

**Table 1:** Demographic characteristics of the total sample and split according to autism diagnosis.

	Total Sample (n = 211)	ASC Group (n = 100)	non-ASC Group (n = 111)	Group differences
**Sex**				*χ^2^* (2) = 1.39, *p* = .498
Male	133 (63.00%)	61 (61.00%)	72 (64.90%)	
Female	74 (35.10%)	36 (36.00%)	38 (34.20%)	
Diverse	4 (1.90%)	3 (3.00%)	1 (0.90%)	
**Age**	28.00 (18 – 62)	28.00 (18 – 62)	27.00 (18 – 60)	*z* = - 0.38, *p* = .707
School type*				*χ^2^* (4) = 1.91, *p* = .752
University	36 (17.10%)	19 (19.00%)	17 (15.50%)	
Gymnasium	76 (36.00%)	38 (38.00%)	38 (34.50%)	
Realschule	51 (24.20%)	23 (24.00%)	27 (24.50%)	
Mittelschule	42 (19.90%)	17 (17.00%)	25 (22.70%)	
No degree	6 (2.80%)	2 (2.00%)	3 (2.80%)	
**Non-verbal IQ**	104. 70 (15.25)	105.95 (13.94)	104.03 (15.75)	*z* = - 0.90, *p* = .369
**Verbal IQ**	104.75 (13.26)	106.47 (12.87)	103.41 (13.39)	*z* = - 1.62, *p* = .106
**Number of Books**				*χ* ^2^(2) = 6.91, *p* = .032
Less than 1 a month	118 (60.80%)	56 (62.20%)	62 (59.60%)	
Between 1 and 2 a month	50 (25.80%)	17 (18.90%)	33 (31.70%)	
More than 3 a month	26 (13.40%)	17 (18.90%)	9 (8.70%)	
**Short-Story Task**				
Comprehension Score	9.40 (1.2)	9.42 (1.17)	9.39 (1.09)	*z* = - 0.64, *p* = .523
Mentalizing Score	8.03 (2.83)	7.81 (2.86)	8.23 (2.81)	*z* = - 1.02, *p* = .309
Spontaneous Mentalizing	28 (13.30%)	9 (9.00%)	19 (17.10%)	
**Adult Asperger Assessment**	8.32 (3.11)	10.66 (2.56)	6.22 (1.79)	*z* = - 10.57, *p* <.001
**Autism Quotient**	34.48 (8.85)	37.36 (8.26)	31.88 (8.59)	*z* = -5.22, *p* <.001
**Empathy Quotient**	20.98 (11.72)	17.05 (10.43)	24.55 (11.74)	*z* = - 4.96, *p* <.001
**Alexithymia**	46.08 (9.78)	46.94 (9.68)	45.29 (9.85)	*z* = - 1.29, *p* = .198
**Sensory Inventory (difference score)**	3.81 (6.05)	5.69 (5.54)	2.11 (6.00)	*z* = - 4.42, *p* <.001

*Gymnasium (higher level education, 8 to 9 years of school after 4 years of elementary school, terminating with the general university entrance qualification), Realschule (intermediate secondary school, 6 years of school after 4 years of elementary school), Mittelschule (9 years of elementary school); ASC, autism spectrum condition group; no-ASC, group without autism spectrum condition; IQ, intelligence quotient.

### 3.1 Short-Story Task

The SST showed an internal consistency of α = .65 for the total score and.27 and.63 for comprehension and mentalizing respectively. There was no significant difference between males and females on the SST (*z* = -1.20, *p* = .232), and no correlation with age could be observed (*τ* = .02, *p* = .767). The SST mentalizing score did not correlate with any of the other autism measures (all p >.05) with the exception of the EOT subscale of the TAS-26 (*τ* = -.13, *p* = .010), which did not remain significant after FDR-correction.

#### 3.1.1 Receiver operator characteristic analyses

An ROC curve was generated to evaluate the SST’s effectiveness in distinguishing autistic individuals from those who are not autistic using the comprehension and the mentalizing score of the SST. The ROC curve for SST comprehension yielded an AUC of 0.48 (95% CI [0.44, 0.60], p = .604), reflecting a poor level of discrimination. The ROC curve for SST mentalizing had an AUC of 0.54 (95% CI [0.38, 0.54], p = .311), also indicating poor discriminative ability.

#### 3.1.2 Predicting SST mentalizing performance

A multivariable regression was computed across the whole sample to predict the SST mentalizing performance as dependent variable based on the independent variables verbal IQ, non-verbal IQ, SST comprehension score, SST spontaneous mentalizing score, level of education, number of books read per month and group assignment (ASC, no-ASC). The model was significant (F(177, 7) = 6.61, *p* <.001) and explained 21.00% of the variance in SST mentalizing performance. Only group membership (ASC, no-ASC) emerged as a significant predictor of mentalizing performance (see **Table 2**).

**Table 2:** Multivariable linear regression predicting mentalizing performance and exploratory binominal logistic regression predicting group assignment (ASC, no-ASC).

Dependent Variable	Predictor	*B*	SE	β	*t*	*p*
SST Mentalizing	Education	0.24	0.23	0.09	1.03	.304
	Verbal IQ	0.04	0.02	0.17	1.70	.091
	Non-verbal IQ	0.03	0.02	0.14	1.56	.121
	Number of books read	0.25	0.27	0.06	0.92	.359
	Spontaenous mentalizing	1.11	0.63	0.12	1.78	.077
	SST Comprehension	0.31	0.19	0.13	1.69	.092
	ASC diagnosis	-0.80	0.39	-0.14	-2.04	.043
Dependent Variable	Predictor	*B*	SE	Exp (*B*)	Wald	*p*
ASC diagnosis	Sex	0.11	0.32	1.12	0.12	.728
	Age	-.00	0.01	1.00	0.01	.937
	Education	0.10	0.19	1.10	0.27	.603
	Verbal IQ	0.02	0.02	1.02	1.44	.231
	Non-verbal IQ	-0.00	0.01	1.00	0.00	.970
	Number of books read	.12	0.23	1.13	0.29	.592
	Spontaneous mentalizing	-0.83	0.56	0.44	2.21	.137
	Comprehension Item 5: “The story is called ‘The End of Something.’ What is the title referring to?”	-1.45	0.47	0.24	9.68	.002

ASC, autism spectrum condition group; no-ASC, group without autism spectrum condition; SST, Short-Story task; IQ, intelligence quotient.

#### 3.1.3 Predicting ASC/no-ASC group assignment

A binominal logistic regression was computed to predict group assignment (ASC, no-ASC) as dependent variable based on the independent variables verbal IQ, non-verbal IQ, SST comprehension score, SST mentalizing score, SST spontaneous mentalizing score, level of education, age, sex and number of books read per month. The model was not significant (*χ^2^* (9) = 10.81, *p* = .289, Nagelkerke *R^2^* = 0.08).

#### 3.1.4 Exploratory analyses

In additional analyses, we examined group differences on specific SST items using χ² tests. The ASC group scored significantly lower on the fifth comprehension item (*χ*² = 11.80, p = .003), which asks, ‘The story is called “The End of Something.” What is the title referring to?’ The correct answer requires recognizing that the title refers to the end of a romantic relationship within the story, rather than simply the conclusion of fishing together. All other questions showed no significant differences between the two groups (*p* >.05).

In a second additional analysis, we repeated the binominal logistic regression above to predict group assignment, choosing the fifth comprehension item as it was the only item to show a significant group difference. The model was significant (*χ^2^* (8) = 20.99, *p* = .007, Nagelkerk2e *R^2^* = 0.14), predicting 65.90% of cases correctly. The sole predictor of group assignment was the fifth item of the comprehension scale (see **Table 2**).

## 4 Discussion

The aim of this study was to evaluate the discriminative power of the Short-Story Task (SST) in a complex clinical setting, specifically among 211 outpatients undergoing autism diagnostics at a clinic and polyclinic for psychiatry and psychotherapy. We hypothesized that the SST would effectively differentiate between autistic and non-autistic individuals, thereby enhancing the accuracy and reliability of autism diagnostics in outpatient services.

Our study revealed significant group differences in established autism measures, such as the Adult Asperger Assessment (AAA), Autism Quotient (AQ), Empathy Quotient (EQ), and Sensory Inventory (SI). While these tools provide valuable insights, it is important to interpret the results with care. For instance, research shows that self-report questionnaires like the EQ rely strongly on mental imagery (38), which can be reduced in autistic individuals (39) and potentially resemble low empathy regardless of actual empathic abilities. Similarly, the AQ, which relies on self-perception, may introduce discrepancies between self-reported traits and actual behaviors (40). To ensure diagnostic accuracy, a comprehensive assessment process was employed, incorporating psychological testing, structured interviews, and external anamnesis, followed by multidisciplinary team discussions.

No significant differences were observed in SST performance between the groups. Importantly, the SST was not used as a diagnostic tool in this study, which avoided potential biases in group assignment. While this approach allowed for an unbiased comparison of SST performance, it also limited our ability to directly compare the SST with other diagnostic measures, as these measures, along with structured and unstructured diagnostic interviews, determined group membership. The lack of significant differences in SST performance suggests that the SST may not be sensitive enough to identify autism based solely on mentalizing performance scores. This conclusion is supported by the SST’s moderate internal consistency and its poor discriminative ability between ASC and non-ASC groups, as indicated by ROC analyses.

Although the SST has previously demonstrated good discriminatory ability in distinguishing between autistic and non-autistic individuals (22), and between autistic and depressed individuals (23), its effectiveness appears diminished in a sample with various comorbidities (10, 13). Individuals in our sample may have developed camouflaging techniques and exhibited higher alexithymia, both of which are associated with complications during the diagnostics process (9, 17). Moreover, although the significant correlation between the externally oriented thinking scale of alexithymia and SST mentalizing performance did not hold after correction, it may still indicate a weak relationship between reduced attention to one’s own emotions and the ability to infer feelings and intentions of non-autistic others or characters within a story.

Regression analysis revealed that group membership (ASC vs. non-ASC) was a significant predictor of SST mentalizing performance. This suggests that, while the SST may not strongly differentiate between ASC and non-ASC groups overall, certain elements of the task do capture underlying differences in mentalizing abilities associated with autism, particularly when mentalizing is applied to non-autistic individuals and characters. However, SST comprehension and mentalizing scores were not effective in directly predicting group membership, highlighting a limitation in their diagnostic utility.

Notably, exploratory analyses uncovered that the ASC group performed significantly worse on a specific comprehension item involving the understanding of a romantic relationship’s end within the story. We are aware that exploratory findings have to be interpreted with caution. Nevertheless, this finding may suggest that certain narrative elements or themes may be particularly challenging for individuals with ASC, potentially due to difficulties in processing social and emotional cues, especially when they are not prompted (41). A subsequent logistic regression using this specific item as a predictor effectively distinguished between ASC and non-ASC groups, suggesting that individual replies to SST items, especially those tapping into complex social or emotional understanding, may offer greater discriminatory power than the overall SST scores.

While one might consider the possibility that autistic individuals may have deficits in linguistic comprehension, we found no significant differences in the overall comprehension score or in the other comprehension items between the two groups. Additionally, there were no differences in verbal or non-verbal IQ between the ASC and non-ASC groups, further suggesting that purely linguistic factors are unlikely to explain these findings.

Interestingly, although the item focusing on the story’s theme (a break-up) was not originally classified as a mentalizing item, its effectiveness in distinguishing between groups highlights a broader issue. If nuanced aspects of the story’s break-up are not fully recognized, the comprehension question may shift from measuring detailed understanding to evaluating a broader grasp of subtext. This suggests that comprehension items might measure overall interpretive ability rather than just specific comprehension skills.

These insights suggest that while traditional diagnostic tools still rely on specific cut-off scores, there may be value in considering a shift from focusing solely on these thresholds to evaluating the reasoning behind individual responses in the SST. By examining how individuals interpret social narratives and characters’ actions, this approach could provide a more detailed understanding of cognitive and emotional processing. Rather than emphasizing predetermined thresholds, it allows for a more nuanced view of how individuals articulate their understanding, which could offer insight into their cognitive and emotional experiences. Additionally, since the SST is not a self-report measure but assesses actual interpretive responses, it could provide a more direct reflection of real-world understanding, particularly in social contexts. While the current analysis follows the SST manual (21), future research could explore whether a more narrative-focused analysis of individual responses might complement traditional diagnostic approaches, potentially enhancing the SST’s diagnostic utility. This approach aligns with a broader trend in autism assessment, emphasizing a move towards personalized, individual profiling that accounts for cognitive diversity within the spectrum (42). Traditional diagnostic tools often rely on scoring thresholds that may not fully capture the complexity of autistic experiences and may inadvertently exclude subpopulations, such as autistic females (43).

While this approach shows promise for enhancing understanding and guiding psychosocial interventions, we acknowledge that further research is needed to establish its potential impact on diagnostic accuracy and utility. This shift toward a more personalized approach should be viewed as complementary to, rather than a replacement for, existing diagnostic practices.

The study has several strengths, including a large sample of individuals seeking autism diagnostics from a specialized outpatient clinic and the SST’s objective assessment format, which is not used for group assignment. The SST’s question-answer approach also provides valuable insights into real-life scenarios and social-emotional processing. However, there are notable limitations.

Firstly, the sample comes from a psychiatric clinic and polyclinic, which means it includes more complex cases with comorbidities. While this sample includes patients that are particularly difficult to diagnose, it could limit how well the findings apply to other groups. Additionally, the high levels of alexithymia observed in the sample, which are associated with difficulties in emotional processing, might have influenced the results. Elevated alexithymia scores could confound SST performance and complicate its interpretation, suggesting that its utility may vary based on the emotional and cognitive characteristics of the sample.

Lastly, research indicates that autistic individuals often benefit from frequent social learning experiences through reading (44), and while our sample’s mentalizing performance was not influenced by the number of fiction books read, the measure used may be too simplistic to capture the full range of reading experiences. This limitation underscores the need for more nuanced, research to better understand how social and emotional processing evolves over time in autism.

## 5 Conclusion

In conclusion, this study evaluated the discriminative power of the Short-Story Task (SST) in a complex clinical setting with individuals seeking autism diagnostics. While the SST did not differentiate between autistic and non-autistic groups, our findings suggest that its diagnostic sensitivity may be limited in populations with various comorbidities.

However, certain narrative elements, such as social and emotional cues, showed potential for distinguishing between groups. This suggests that a narrative-focused approach, which is not inherent to the SST but could be explored further, may offer more valuable insights into individual cognitive and emotional processing. This aligns with a broader trend in autism diagnostics toward moving beyond rigid thresholds and considering individual profiles.

While the SST is not designed to specifically evaluate narrative responses, integrating a more narrative-focused analysis could complement traditional assessments and provide a more nuanced understanding of autism. Further research is needed to explore how this approach might enhance the SST’s diagnostic utility, particularly in complex clinical populations.

## Data Availability

The raw data supporting the conclusions of this article will be made available by the authors, without undue reservation.
